# No. III. On Medico-Legal Evidence in Cases of Insanity

**Published:** 1854-07-01

**Authors:** Forbes Winslow


### LETTSOMIAN LECTURES.

### No. III.

### ON MEDICO-LEGAL EVIDENCE IN CASES OF INSANITY.

*Delivered before the Medical Society of London.*

By FORBES WINSLOW, M.D., D.C.L.

I HAVE selected for my third and concluding Lecture a subject of great, vast, and of daily increasing magnitude and importance, not only if viewed in relation to our position as citizens of the state, privileged to live under the protection of a monarchical government and a constitutional sovereign, but as members of an honourable and learned profession, exercising—rightly, justly, and advantageously exercising—an incalculable degree of moral influence through all sections of society, and all departments of art, science, and philosophy. Is it possible to over-estimate or to exaggerate the interest of the subject now under consideration? I propose to submit to your critical judgment a sketch of our high and responsible vocation as medico-legal witnesses—to suggest for adoption certain general principles of evidence, which may serve as our guide when called upon to give testimony in difficult, doubtful, and disputed cases of insanity. The position of the medical witness, even under the most favourable circumstances, is perplexing, anxious, and embarrassing. The character of his education, the peculiarity of his habits of thought, the philosophic cast of his mind, his constant and earnest search after truth, the nature of his daily professional occupation, ill adapt him for contending in the forensic arena with the knowledge, ability, and subtle acumen which are so often brought to bear (in courts of justice) against those little skilled in the art of legal fence. Occasionally we have to give testimony in relation to matters of *fact;* to describe *physical* states—phenomena cognizant to sense. For example: in cases of sudden death from supposed poisoning, the toxicologist has certain well-defined scientific data to guide him to a right conclusion; he is in possession of well-recognised tests, which bring him almost unerringly to a sound and safe deduction; his evidence has reference more to an *exact*, than to a speculative—to a certain, than an uncertain science; his province (when in court) is simply to record the results at which, after careful investigation, he has arrived. The questions involved in the inquiry, whether death, under suspicious circumstances, was natural, self-inflicted, or the effect of extraneous violence, are not *necessarily* intricate, obscure, or difficult of satisfactory solution. How different, however, is the position of the witness, when his mind is brought to the consideration of questions connected with morbid mental phenomena? In these exalted inquiries he has no fixed or certain test—no infallible standard—no well-defined rules—no principles of exact science, to aid him; no beacon to protect him from the rocks and quicksands which beset his course—no chart to refer to in times of difficulty—no compass to guide him in the hour of danger—no harbour of refuge into which he can run his fragile vessel when the tempest is howling and destruction impending. As medico-legal witnesses, the obstacles with which we have to contend are often of a grave and serious character. We have to deal with phenomena, of the essence or intimate nature of which we know absolutely—positively, nothing. It is our duty to elucidate principles of belief—to unravel motives of action—to explain erratic conduct the most anomalous and extraordinary; we have to trace the line which separates passion—the subtle and shifting transformations of wild, ungovernable, and impetuous passion—from the excitement of mania, and the morbid emotions incident to the minor forms of diseased mind; to sketch the varying frontier, the nice and shadowy distinctions, which separate lunacy from malignity—madness from brutality; to point out where folly merges into mental derangement—where *responsibility* terminates, and *irresponsibility* commences; to distinguish between *eccentricity* and *insanity*—*crime* and alienation of mind—*vice* and *mental derangement*—between the delusions of the lunatic and the false conclusions—the illogical deductions—the unphilosophical reasoning of men of sound intellect and of rational understanding,—to separate the normal rhapsodies of the healthy imagination, and the Arcadian illusions of the poet, from those *morbid* conceptions of the fancy—those

> ——— "Daggers of the mind—false creations
> Proceeding from the heat-oppressed brain"—

those "thick-coming fancies," the products—the well-recognised, indisputable symptoms of a mind thrown off its healthy balance by *actual cerebral disease.*

There is no possibility of our placing the diseased mental elements submitted to our critical examination in a psychological crucible or test-tube; we cannot avail ourselves, in these delicate investigations, of the aid of the microscope; there is no mode by which we can penetrate behind the curtain, or tear aside the veil that divides the *material* from the *immaterial—mind* from *matter;* there is no possibility of our obtaining access to that mysterious chamber where the spiritual portion of our nature is elaborated; we have no gauge, no square rule, by which we can ascertain in all cases, with any approach to chemical or mathematical accuracy, an accurate idea of the actual condition of the mind, when apparently under a cloud. In the elucidation of these points, we are in a great measure left to our unaided mental sense—to the uncertain guidance of our own deceptive experience, and alas! often, fallible judgment.

We enter the witness-box, charged, under the solemn sanction of an oath, to decide the important questions as to the legal and moral responsibility of our fellow-men. In capital cases, we are called upon to declare whether the criminal was or was not insane when he committed the act; whether, by disordered mind, he was reduced to a state of legal irresponsibility. In other cases, equally important matters are submitted to our adjudication, involving points relative to the competency of persons to make testamentary dispositions of their property, or manage, during life, themselves and their affairs. In the former case, the life of a fellow-creature is made contingent upon the evidence of those deputed to examine him, and delegated with the responsibility of recording their medico-legal opinion as to his state of mind; in the latter instance, we are expected to depose to the competency of certain persons to exercise the otherwise inalienable privilege of disposing of property agreeably to their own notions of the law of inheritance and conceptions of what is just; and, in the third case, it is our province to decide, not upon the solemn question of life or death, but whether a fellow-citizen is in a condition of mind to justify the law in alienating from him his *civil rights,* depriving him of the control of his person and affairs, and destroying, by a legal declaration of lunacy, his free and independent agency. In the first case, it is our imperative duty to avert, if possible, *actual* death—a death of *moral* ignominy and of *physical* suffering; in the latter instance, it is left for us to pronounce whether legal dissolution is to be recorded against the party whose mind is the subject of medico-judicial inquiry. In the former case, it may, happily, be in our power to rescue a fellow-creature from the scaffold; and, in the latter instance, we may, by our evidence, have the not less pleasing gratification of shielding him from the expensive, but nevertheless, under proper circumstances, humane guardianship of the Court of Chancery.

Under circumstances like those I have now cursorily sketched, we have, as may readily be conceived, to contend with serious impediments. The witness has to encounter the prejudices and ignorance of those by whom he is surrounded—of those who, if otherwise enlightened, are too disposed to forget that the mental conditions relative to which he has to speak are the exceptions to the general laws by which human nature is guided, and that they can only be elucidated by facts of an extraordinary character, which rarely present themselves in the state of society in which an individual exists. In attempting to give the court before which he is subpœnaed a lucid statement of his opinion, based upon actual experience, long-continued observation, reflection, and patient study, the views thus expounded are too often considered either as the offspring of a false philosophy—a mawkish sensibility—a distorted science—the affectation of a learned and metaphysical subtlety—or, alas! as the sordid result of the paltry *honorarium* awarded to him for the expression of his professional opinion! The medical witness has to encounter the sarcastic doubts, the special pleading, the suspicious inuendoes, the legal finesse, of the acute and accomplished advocate, always on the alert to perplex and confound him; he has also arrayed against him the unbending dicta of the judge, and inexperience of the jury, easily misled by the plausible appeals, the persuasive eloquence, and *ad captandum* arguments of the counsel, who, occasionally, in the discharge of his duty as an advocate,* considers

* Lord Brougham, in his celebrated speech on Queen Caroline's trial, thus describes the duty of an advocate:—" An Advocate, by the sacred duty of his connexion with his client, knows, in the discharge of that office, but one person in the world—that client, and none other. To save that client by all expedient means—to protect that client at all hazards and costs to all others, and among others, to himself, is the highest and most himself justified, whilst defending the interests of his client, to combat truth by sophistry—to dumb-found, confuse, and entrap the witness—dazzle and bewilder the judge—hoodwink the jury, and, by a combination of qualities which the accomplished *nisi prius* advocate and practised disputant is so competent to call into successful operation, make the "worse appear the better reason," pervert the ends of truth, and thus make what ought to be revered as the TEMPLE OF JUSTICE, and held sacred as the MAJESTY OF THE LAW, a mockery and a jest.*

Having referred to the peculiar position of the medico-legal witness, I would direct attention briefly to the value—special and peculiar value—of the testimony of those who have directed their attention almost exclusively to the study of medical psychology, and who, by patient investigation and long experience, have obtained a practical insight into the characteristics of the varied phenomena of mental alienation, the habits and peculiarities of the insane, and who are therefore peculiarly fitted to give evidence in these cases.

Questions of great difficulty and complexity often arise in the course of important judicial investigations, involving matters of science, upon which the judge, jury, and counsel are incompetent, from actual want of knowledge, to form a sound and accurate unquestioned of his duties; and he must not regard the alarm, the suffering, the torment, the destruction, which he may bring upon others. Nay, separating even the duties of a patriot from those of an advocate, he must go on reckless of consequences, if his fate should unhappily be to involve his country in confusion!"—(Oct. 3, 1820.)

* "Addresses to a court of justice or a judicial body by a paid advocate, although they tend to a practical conclusion, do not fall under the head of *deliberative oratory*. The advice is not given upon the personal security, credit, and authority of the speaker, nor is he understood to speak his own convictions, but merely to follow his instructions, and to present the facts of the case and the application of the law to it, in the light most favourable to his client. Hence a paid advocate speaks without moral weight, and his arguments merely pass at their intrinsic value, without deriving any additional force from the source from which they proceed."—*An Essay on the Influence of Authority in Matters of Opinion*, by George Cornewall Lewis, Esq., p. 132.

Sir James Johnston happened to say that he had no regard for the arguments of counsel at the bar of the House of Commons, because they were paid for speaking. *Johnson.* "Nay, sir, argument is argument; you cannot help paying regard to their arguments, if they be good. If it were testimony, you might disregard it, if you knew it were purchased." There is a beautiful image in Bacon upon the subject: "Testimony is like an arrow shot from a long-bow—the force of it depends on the strength of the hand that draws it; argument is like an arrow from a cross-bow, which has great force though shot by a child."

judgment. With a view to their elucidation, men of repute, termed in France *experts*, and in Italy *periti*, who have made the matter at issue a special object of study, are called upon for their testimony, and their evidence is generally considered as final and conclusive. In a case in which it is necessary, in order to satisfy the ends of justice, to submit certain portions of food, or the contents of the stomach, to careful chemical analysis, in order to ascertain, by the aid of delicate tests, whether a person had come to his death by fair means, professional gentlemen who have a reputation for having paid particular attention to such investigations, and who are practical and experienced chemists and toxicologists, are called upon for their opinion, and upon the result of their investigations the life or death of a fellow-creature often depends. No reasonable man disputes the value of such testimony.* A similar course is pursued when any difficult and complicated question arises connected with navigation, mechanics, or civil engineering. The most able men of the day are summoned to solve knotty points, and to settle questions of disputed science, which sagacious and experienced minds are only able satisfactorily to determine. For what object are matters of great difficulty and doubt submitted to the adjudication of the judges assembled in the highest courts in the kingdom, if it were not to obtain from men, presumed by their elevated station to possess the *maximum* amount of legal lore, a safe and satisfactory opinion?† If we were not daily in the habit of deferring to the knowledge and judgment of expe-

* "For all purposes of philosophical observation, a knowledge of the proper science and a peculiar training of the senses are requisite, and, therefore, a witness who possesses these qualifications is far more credible than one who is destitute of them. For example, a scientific naturalist, who reports that he has seen an undescribed animal or vegetable in a remote country, is far less likely to be mistaken than a common traveller, ignorant of natural history. A skilled witness of this sort may be considered in a certain sense, as a *witness of authority*, inasmuch as his previous study and habits of observation give a peculiar weight to his report of the phenomenon."—*Essay on Authority in Matters of Opinion*, by G. Cornewall Lewis, Esq.

† Cicero, in enumerating the circumstances which give authority to testimony, places first, *virtus*, and afterwards, *ingenium*, *opes*, *ætas*, *fortuna*, *ars*, *usus*, *necessitas*, and sometimes *concursio rerum fortuitarum*. With regard to the latter, he says, " Sed reliquis quoque rebus, quanquam in iis nulla species virtutis est, tamen interdum confirmatur fides, si aut ars quædam adhibetur (magna enim est vis ad persuadendum scientiæ) aut usus; plerumque enim creditur iis qui experti sunt."—*Topica*, c. 19.

rienced and intelligent minds, why should there exist any necessity for the establishment, in connexion with the judicature of this country, of courts of appeal? Does not the eminent *common* law barrister bow with great submission to the distinguished *equity* counsel, and willingly and implicitly refer to *his* decision matters of great complexity connected with his own department of the profession? Is not the learned body of British jurists divided and subdivided into sections, each having its distinct and separate court? An analogous practice is adopted in our own science, and we are repeatedly availing ourselves of the superior attainments and practical knowledge of those whom we know have acquired a large amount of experience in special departments of our profession. I cannot conceive why medical men, who have devoted themselves to the study of the diseases of the mind, should not be equally competent with the experienced mechanist, the practical engineer, the learned jurist, the scientific chemist, and the toxicologist, to pronounce *ex cathedrâ* on points coming strictly within their own peculiar province.*

Whilst upholding the testimony of able, scientific, and experienced men, I would protect myself from the imputation of urging a slavish or blind submission to men even of admitted acute and vigorous intellects. "Although," says Lord Bacon, "the position be good, *oportet discentem credere*, yet it must be coupled with this, *oportet edoctum judicare;* for disciples do owe unto masters only a temporary belief, and a suspension of

* "In order that a person should be eminent in a learned profession, it is necessary that he should combine a knowledge of its principles with that judgment, tact, dexterity, and promptitude of applying them to actual cases, which are derived from habits of practice. The like may be said of persons conversant in the constructive arts, as architects and engineers, of the military and naval services, of agriculturists, gardeners, manufacturers of different kinds, &c. In order that they may give sound advice with respect to any practical question belonging to their own department, it is necessary that they should combine actual experience with abstract knowledge. In some cases, that experience implies even manual skill, which can only be acquired by practice. For example, a surgeon would not be a competent judge on a question of practical surgery, unless his judgment were assisted and corrected by actual manipulation of his instruments. In like manner a person cannot be a competent judge of works of art, such as statues, pictures, coins, engravings; or of articles of trade, as horses, wines, plate, &c., without practical observation and experience. In these cases a certain training of the sight is necessary, analogous to the training of the hands and limbs in a mechanical employment or trade requiring bodily dexterity."—G. Cornewall Lewis, *On the Marks of Trustworthy Authority, in an Essay on the Influence of Authority in Matters of Opinion.*

their own judgment until they be fully instructed, and not an absolute resignation or perpetual captivity."*

When a medical man is summoned to record his testimony in a court of law, upon a case in which it is important to ascertain the degree of sanity that existed at any stated period, he gives his opinion to the best of his knowledge and ability, upon an *abstract* point, without any reference to ulterior results. He has not to regard the legal consequences of his evidence; it is not for the witness to consider whether life is to be prolonged to an indefinite period, or whether a fellow-being shall be immediately launched into eternity. To the questions—"Do you consider the party insane?—was he so, according to the best of your judgment, at such a period?" the medical gentleman experienced in the characteristics of insanity answers, negatively or affirmatively. If the accused party escape punishment, as the result of his opinion—if, in consequence of the medical evidence, his life be saved—I do not see by what right he can be held up to public odium and censure. The witness is not to be considered responsible for the operation of the laws (be they good or bad), neither is he accountable for the escape of the prisoner, if acquitted on the plea of insanity, and thereby exempted from the extreme penalty awarded for his crime. The witness is sworn to state the truth according to his honest convictions, regardless of the *legal* results of his evidence.

There is, alas! a disposition in cases of alleged insanity to repudiate in courts of law all evidence of this specific and scientific character. I am bound, in justice to the legal profession, to confess, that, occasionally the evidence of medical men adduced at inquiries of this nature is extremely unsatisfactory. It is too often the practice to place in the witness-box professional men wholly incompetent to give testimony in cases of disputed insanity;—incompetent, from ignorance of the meaning of the ordinary medical terms used to designate the recognised forms of diseased mind, as well as from inexperience in the precise bearing of medico-legal evidence. I have, in my time, seen men manifesting great self-assurance and unbounded confidence in their own knowledge and sagacity, step flippantly and eagerly into the witness-box, only

* *Adv. of Learning*, b. I. vol. i. p. 45.

to retire sadly mortified. It has been my duty to see some melancholy exhibitions of painful professional humiliation, and I must admit, that in most cases they have arisen from an actual want of information on the subjects upon which the witnesses have been examined! If I were not indisposed to descend to particulars, I could refer to several recent trials for illustrations of what I have said. It is too commonly imagined that a knowledge of insanity comes by intuition, and that, without special and particular investigations of this class of affections, any well-informed and regularly-educated medical man is qualified to give evidence in courts of justice upon these matters. This is a sad mistake; but, unfortunately, the discovery is rarely made until the medical man has recorded his testimony.

The illiberal feeling to which I have adverted, as exhibited towards those who, in the discharge of an anxious and solemn duty, are occasionally called upon to give evidence in relation to insanity, has, on more than one occasion, manifested itself in our courts of judicature.

A few illustrations will suffice. The Lord Chief Justice of England, when playfully (I presume) alluding, in the celebrated Bainbrigge case, tried at the Stafford Assizes, to the evidence of the three physicians who had recorded their opinion in favour of the insanity of the testator, observed after they had retired from the witness-box, "The medical men who have just been examined need not be detained any longer?" Mr. Keeting—"Certainly not, my lord;" and upon Sir A. Cockburn assenting, Lord Campbell remarked, "Let it be fully understood, on both sides, that the medical men may take their departure;" and, addressing the three physicians, his lordship continued, "*You may go home to your patients, and I wish you may be more usefully employed there than you have been here!*" Again, in his charge to the jury, fearful that his graceful compliment might be obliterated from the recollection of the court, Lord Campbell added (when analyzing the medical testimony), "*We have had, during this trial, the evidence of three medical witnesses, and I think they might as well have stayed at home, and have attended to their patients.*"*

In connexion with these discursive sallies—these extra-judicial

* Taken from a report of the trial, printed for private circulation, from the short-hand writer's notes.

pleasantries (for such I presume they must be considered)—it is necessary to associate the subjoined facts:—This remarkable and celebrated trial was one of the most important disputed will cases which has been made the subject of litigation, in this country, for a considerable period; upon its issue depended property to a vast amount; the investigation of the facts of the case occupied more than a week; and some of the most illustrious advocates and distinguished common and equity lawyers were retained as counsel. The question at issue rested entirely upon the sanity or insanity of the testator. Evidence of a very conflicting character was adduced; the facts in relation to the alleged insanity were strangely contradictory; and it was therefore deemed necessary to bring specially from London, three physicians, who were, I presume, considered to be men of experience, sagacity, and science, to hear the sworn testimony; and, as *experts,* to state, to the best of their judgment, basing their conclusions upon the evidence adduced in court relating to the testator's condition of mind, whether he, when the will was executed, was of a healthy, sound, and disposing intellect. Can we conceive a more important and relevant question for the medical witnesses to decide, and one coming more legitimately and strictly within their jurisdiction?*

In March, 1848, the following case occurred:—A woman was delivered of a child. On the 10th of December, at the expiration of a week, she was seized with a violent attack of puerperal mania. Mr. Bell, of Tilstead, her medical attendant, gave instructions that she should be carefully watched, and on no account to have access to her child. On the 23rd of December, in the absence of her attendant, she persuaded her daughter to bring the infant to her, and obtaining possession of a razor, she almost immediately cut the child's throat! The prisoner appeared quite calm and collected after the occurrence; she admitted that she had destroyed the child, and that the crime was premeditated. The medical witness, in answer to a question from Lord Denman, before whom the case was tried, very properly declared, that the prisoner might have known that she was going to kill the child;

* In this case the jury returned an unanimous verdict against the will, on the ground of insanity. Owing to some informality, the case was to be tried a second time at Stafford, and two of the former medical witnesses were subpœnaed to give evidence; but, by mutual consent, the will was declared invalid, and consequently the question did not go to trial.

and he was of opinion that she acted under a sudden and uncontrollable impulse. The judge took objection to the term "sudden," because the prisoner had deliberately asked for her child, and had permitted a quarter of an hour to elapse before the razor was solicited. Mr. Bell then, with great propriety, observed, that the act was committed under an uncontrollable impulse, acting upon a mind previously diseased. In his charge to the jury, Lord Denham is reported to have said, that he was of opinion, "That the judgment of the medical gentleman had been very rashly formed." "How," exclaimed his lordship, with marvellous simplicity, "*How could one person dive into the mind of another, and express an opinion with regard to its being in an unsound state, when there was no evidence of any alteration of conduct, or any circumstances in the case to show alienation of mind?*"

If the act itself was insufficient to establish the insanity of the unhappy woman, her *prior* morbid condition—viz., that of *puerperal insanity*, (a form of mental derangement so generally associated with an intense and uncontrollable desire to destroy the offspring)—ought to have satisfied a judge fitted to adjudicate in these important cases, that the evidence adduced was *amply sufficient to justify the acquittal of the prisoner.* Lord Denman, however, thought otherwise.*

Leaving this section of the subject, I now proceed to the con-

* In a case of "wilful fire-raising," tried in Edinburgh some years back, the plea of insanity was raised in behalf of the prisoner. The presiding judge was the Lord Justice Clerk. Medical men of great eminence gave evidence in favour of the culprit; but in consequence of the prisoner "showing a certain degree of cunning and address" during the trial, the judge held that his mind was not in a state to exempt him from legal responsibility; and he laid it down that "this was just one of those cases in which the jury was a better judge of the real state of a man's mind, from hearing all the facts connected with the crime charged against him, than medical men who only saw the prisoner once or twice, when he might be cunning enough to perceive the object of their visit, and carry through a deception upon them for the express purpose of finding what the medical man would be led to think of him." In consequence of his lordship thus throwing aside the medical testimony, a verdict of *guilty* was recorded against the prisoner, and he was sent to Millbank prison preparatory to transportation. Whilst in prison his insanity immediately manifested itself; and it was deemed advisable to send him as a lunatic to Bethlehem hospital. This is but one illustration out of many I could cite, showing the folly of repudiating medical evidence in cases of insanity, and the absurdity of supposing that a jury, however intelligent, and a judge, however conscientious and sagacious, are competent, in questions of this kind, without the aid of scientific medical testimony, to arrive at a right conclusion.

sideration of the principal points involved in this inquiry, premising that I have, in unison with gentlemen of great eminence, of high standing at the bar, as well as with distinguished men connected with our own department of science, had to deplore—to deeply lament—the absence of any approximation to uniformity of opinion; to regret the want of well-defined and settled *first principles* to regulate our judgment, and serve as beacons, when summoned into courts of justice to record our opinion upon questions of such scientific, social, and national importance. Upon these occasions, how often medical witnesses are conscious of the want of some specific rules to which they could appeal in cases of complexity, of doubt, difficulty, and obscurity! It is with the object of illustrating some of these points, and of suggesting a few principles in regard to medico-legal evidence, that I bring this matter before the profession.

The occasions upon which medical evidence is required in courts of law in reference to questions of insanity, and competency to manage the person and property, are as follows:—

1.

CASES IN WHICH THE PLEA OF INSANITY IS URGED IN EXTENUATION OF CRIME.

2.

CASES WHERE ATTEMPTS ARE MADE TO INVALIDATE THE LEGAL OPERATION OF TESTAMENTARY DISPOSITIONS OF PROPERTY, ON THE GROUND OF MENTAL INCOMPETENCY.

3.

WHEN LEGAL PROCEEDINGS ARE INSTITUTED TO INVALIDATE A MARRIAGE CONTRACT ON THE PLEA OF INSANITY AND IMBECILITY.

4.

IN COMMISSIONS "DE LUNATICO INQUIRENDO," ISSUED BY THE LORD CHANCELLOR, WITH THE VIEW OF ASCERTAINING THE EXISTENCE OF UNSOUNDNESS OF MIND, AND COMPETENCY OF THE PARTY (THE SUBJECT OF INVESTIGATION) TO MANAGE HIS PERSON AND PROPERTY.

5.

CASES IN WHICH MEDICAL MEN ARE CALLED UPON TO CERTIFY TO THE EXISTENCE OF INSANITY, JUSTIFYING AN INTERFERENCE

WITH THE PERSON OF THE LUNATIC, AND DEPRIVING HIM OF HIS FREE AGENCY, EITHER FOR THE PURPOSE OF PLACING HIM UNDER TREATMENT, OR PROTECTING HIM FROM THE COMMISSION OF ACTS OF VIOLENCE TO HIMSELF OR OTHERS.

If we refer to the acknowledged legal text-books upon the "Law of Lunacy;" if we examine the recorded opinions of all the distinguished jurists who have adorned the bench; if we wade through the published decisions of eminent criminal and ecclesiastical judges; if we (as a last resource) appeal to the combined wisdom and elaborate judgment of the learned conclave delegated by the British Parliament in 1843, to give an exposition of the law of lunacy, before the highest court of judicature in the country, we are obliged to confess that there exist no settled, uniform, fixed, or unerring principles of jurisprudence, or legal tests, in regard to questions of criminal or civil insanity.

Analyzing with great care the legal *dicta* of the judges, it would appear that the courts of law have, upon different occasions, admitted the following conditions of mind as evidence of insane and legal irresponsibility:—

1.

AN ABSOLUTE DISPOSSESSION, BY DISEASE, OF THE FREE AND NATURAL AGENCY OF THE MIND; PARTIAL INSANITY BEING NO EXCUSE FOR CRIME.

2.

THE EXISTENCE OF A DELUSION, THE CRIMINAL ACT BEING THE IMMEDIATE AND DIRECT RESULT OF THE MORBID IDEA; THE PROOF OF THE PRESENCE OF A DELUSION HAVING NO POSITIVE AND CLEAR CONNEXION WITH THE ALLEGED CRIME, NOT BEING LEGAL INSANITY, AND NO EVIDENCE OF THE EXISTENCE OF IRRESPONSIBILITY.

3.

A CONSCIOUSNESS OF OFFENDING AGAINST THE LAWS OF GOD AND MAN—IN OTHER WORDS, A KNOWLEDGE OF GOOD AND EVIL.

4.

A KNOWLEDGE OF RIGHT AND WRONG—LAWFUL OR UNLAWFUL—THE PRESENCE OR ABSENCE OF MOTIVE.*

* The judges will not permit the medical witness to infer the existence of insanity from the character of the *act itself*, apart from all other

I cite these *four* points as fairly embodying and truthfully representing the leading and fundamental principles of our criminal jurisprudence. It is unnecessary for me to remind you that occasions have occurred in which the courts have departed—plainly, palpably, and indisputably deviated—from these *dicta*, some of the judges having directed the acquittal of persons arraigned for the commission of capital crimes, in the teeth of the tests laid down in the House of Lords for their guidance. Cases are upon record in which parties have been absolved from legal responsibility who have had no *appreciable* delusion, much less one directly associated with and leading to the commission of the criminal act. Again, "*partial insanity*" has been held as a valid plea. Instances of what are termed "moral" and "impulsive insanity," "transient" and "homicidal monomania," have escaped the web of the law, and have eluded the judicial test. Nevertheless, I have placed before you the recognised and admitted legal criteria of insanity in cases of crime, and it is therefore imperative upon us, from our position, knowledge, and experience, to carefully ascertain whether they are *safe principles* upon which to act in the present advanced state of our knowledge of morbid mental phenomena, and in accordance with the existing enlightened state of the sciences of psychology and jurisprudence.

In considering the first legal test, viz. "*an absolute dispossession, by disease, of the free and natural agency of the mind*"—very little need be said. This condition of mental prostration—of intellectual obscurity—obviously can give rise to no doubt, legal difficulty, or disputation. All must acknowledge evidence of derangement of mind. In the case of Greensmith, tried for murder on the Midland Circuit in 1837, Mr. Justice Parke observed, in his charge:—"Nothing could be more contrary to the law than to infer insanity from the very malignity and atrocity of the crime. It was true, that such crimes could never be committed by men who were in the possession and control of a right reason, and a proper mind; but it was his duty to inform the jury that the complete possession of reason was not essential to constitute the legal, any more than the moral responsibility of man, it being necessary that the party should have sufficient knowledge and reason to discriminate between right and wrong." This may be sound *law*, but it is not sound *psychology*. In many cases the "atrocity and malignity of the crime" afford to the practical physician invaluable evidence of the existence of insanity, the derangement manifesting itself in the character of the *act itself*. I willingly admit that we should cautiously act upon such evidence; but should we not be culpable if we were to set it altogether aside?

the wisdom of exempting this class from responsibility and punishment. In regard to the second point—that of "partial insanity"—the law is thus expounded by the judges. In answer to the question, "If a person under an insane delusion as to existing facts commits an offence in consequence thereof, is he thereby excused?" the reply was as follows: "If the delusion were only *partial*, the party accused was *equally liable* with a person of sane mind. If the accused killed another in self-defence, he would be entitled to an acquittal; but if the crime was committed for any supposed injury, he would then be liable to the punishment awarded by the law to his crime." It will thus be perceived that the law repudiates "partial insanity" as a plea in extenuation of crime, or as an exemption from punishment. In the strictly legal signification of the term, what is "partial insanity?" Lord Lyndhurst clearly defines the condition to be, "a mind in an unsound state—not unsound upon one point only, and sound in all other respects, but that this unsoundness manifests itself principally with reference to some particular object or person." According to this definition, it is evident that "partial insanity" and "monomania" are not, as some have supposed, exactly equivalent terms: they have, however, been so considered by many medical and legal authorities. According to the 64th article of the French penal code, no person, whilst insane, is considered responsible for a criminal act, "Il n'y a ni crime ni délit lorsque le prévenu était en état de démence au temps de l'action." In opposition, however, to this article, M. de Peyronnet, the Advocate-General of France, in the cases of Leger, Feldtmann, and other insane homicides, adopted the view of Lord Hale on this subject, as to the existence of a partial and a total insanity, laying down the principle, that the latter "can alone extricate the criminal from the penalties of the laws." "The distinction between partial and total insanity," he observes, "throws great light on the questions of insanity." In confirmation of this view of the case, he referred at some length to the opinions of Lord Hale, and quoted a passage from his celebrated "Pleas of the Crown." The line of argument, however, adopted by the Advocate-General on these occasions displeased highly the medical jurists of France. M. Georget, who has expressed his astonishment at the *dicta* of Lord Hale, says,

"This writer (Lord Hale) appears professedly to consider property of higher value than human life! There is, then, no excuse for the unfortunate lunatic, who in a paroxysm commits a reprehensible action, even although it should appear to be the result of his particular illusion! and yet the civil acts of this same individual are to be annulled, although they have no relation to the insane impressions which might have influenced his conduct! And even M. de Peyronnet cited such maxims as these with approbation—at least we do not find that he has objected to any of them; all monomaniacs, according to their statements, are liable to become criminals, in spite of the 64th article of our penal code, and may undergo the penalties recorded for atrocious offences."*

I will not, in this stage of the inquiry, consider whether, metaphysically examined, the admission of a "partial" as well as a "general" insanity does not vitiate the psychological theory of the *individuality of the mind*, or the *unity of the consciousness;* but viewing the phrase to import an unsoundness of mind manifesting itself in the form of "monomania," or delusion upon *one prominent point*, the mind being *apparently sane and rational upon all others*, I would ask men of observation and experience, if, in such cases (the most pure and uncomplicated instances that the mind can conceive), the party so clearly and manifestly insane should be held responsible to the criminal law for his conduct, and be treated as if he were of perfectly rational and sane understanding?

Partial insanity no valid excuse—no extenuation for crime! Partial insanity no plea—no justification in criminal cases! How monstrously unphilosophical, how wildly fallacious, how opposed to positive facts, how absurdly illogical, how grossly unjust, how

* "A man may be deranged in his mind, his intellect may be insufficient for enabling him to conduct the common affairs of life, such as disposing of his property, or judging of the claims which his respective relations have upon him; and if he be so, the administration of the country will take his affairs into their management, and appoint to him trustees; but, at the same time, such a man is not discharged from his responsibility for criminal acts. I say this upon the authority of the first sages in this country, and upon the authority of the established law in all times, which law has never been questioned, that although a man be incapable of conducting his own affairs, he may still be answerable for his criminal acts, if he possess a mind capable of distinguishing right from wrong."—*Speech of Attorney-General Sir Vicary Gibbs, on the Trial of Bellingham for the murder of the Right Hon. Spencer Perceval.*

repulsive, how abhorrent to every right-thinking, to every humane mind, and to every christian and philanthropic heart! Apply this judicial, antiquated, and absurd dogma to the great mass of miserable and irresponsible lunatics at this moment legally in confinement, and two-thirds of them would be immediately made amenable to the law for their conduct! If partial insanity can be clearly established, who would be bold enough to declare or define the precise limits of the disease, or to sketch the boundary-line separating a responsible from an irresponsible state of mind? "Where is," we might exclaim, in the language of a distinguished modern historian, when discussing the legality of the resistance made to the tyranny of James II., "where is the frontier where virtue and vice fade into each other? Who has ever been able to define the exact boundary between courage and rashness, between prudence and cowardice, between frugality and avarice, liberality and prodigality? A good action is not distinguished from a bad action by marks so plain as those which distinguish a hexagon from a square."* Who can safely draw the line of demarcation between night and morning, between light and darkness? or say at what precise moment health fades into disease? "Who can mark precisely the frontiers, the almost imperceptible limits, which separate insanity from sanity? Who can number the degrees by which reason declines and falls into annihilation? This would be to prescribe limits to that which is illimitable, to give rules to folly, to be bewildered with order, to be lost with wisdom."†

The existence of *delusion* is the next judicial test and legal plea referred to. "The true criterion" (says Sir John Nicholl), "the true test of insanity, I take to be absence or presence of what, used in a certain sense, is comprehended in a single term—*viz., delusion. In the absence of anything in the nature of delusion, the supposed lunatic is, in my judgment, not properly insane.*"‡ Lord Denman thus gives exposition to the law: "To say a man was irresponsible, *without positive proof of any act to show that he was labouring under some delusion, seemed to him to be a presumption of knowledge, which none but the*

* History of England, by the Right Hon. B. Macaulay, M.P., &c.

† M. d'Aguesseau, before the Parliament of Paris.

‡ Dew *v.* Clarke.

*great Creator could himself possess!"** Such being the law, what, I ask, is the legal definition of this valuable, this fixed, and infallible test? Lord Brougham defines a delusion to be, "*a belief of things as realities which exist only in the imagination of the patient.*" Sir John Nicholl says, "*A delusion is a belief of facts which no rational reason would have believed.*" When speaking of the latter definition, Lord Brougham observes that it is liable to one exception—viz., that it gives a consequence for a definition. His lordship then refers to his own definition, which he declares not to be open to the same objection.

Belief depends upon testimony; and the degree of credence attached to such testimony must necessarily be materially influenced by the nature of the evidence adduced, as well as by the character of the party giving it, and the knowledge, intelligence, and health of the mind, the recipient of it. May not a person believe in the existence of "something extravagant," which exists "only in his imagination," and yet be free from delusion, and of sound and rational mind? A distinguished philosophical writer says, "We obtain experience either by the evidence of our own senses, or by the testimony of others. The testimony of our senses, though generally considered as one of the highest degrees of evidence, is often fallacious and often deceptive. Although the impression is properly made on organs that are in their sound state, yet the ideas conveyed thence to the mind may be so varied and modified by the imagination as entirely to mislead the judgment. Thus every part of natural history, and medicine above all others, is crowded with facts, *attested by eye-witnesses of supposed veracity, which facts, notwithstanding, never had any existence but in their own imagination.*"† A person of sound mind may, upon false, shallow, and inconclusive evidence, believe in the wildest improbabilities, and firmly maintain their truth; nevertheless, who would have the boldness to affirm, that he was under a delusion?‡ Lord Brougham's definition appears to involve exclusively

* Regina *v.* Smith. † Campbell's Philosophy of Rhetoric.

‡ "False and unfounded opinions have been entertained by entire communities without question, for ages. Not merely has this been the case with respect to false religions, and legendary accounts of early history, interwoven with the religious and patriotic feelings of the people, but even the action of the *healthy* imagination. If I were bold enough to commit myself to a definition of the term delusion, I would venture, with submission, to give the following:—*A delusion is a belief in the existence of a something extravagant, which has, in reality, no existence except in the* DISEASED *imagination of the party, and the absurdity of which he cannot perceive, and out of which he cannot be reasoned.* In this definition I think a scientific distinction is drawn between the *eccentric* conceptions of a *healthy*, and the *morbid* creations of a *diseased* imagination and judgment; a principle which we should never lose sight of in our medico-judicial inquiries, definitions, and evidence. By legal authorities, delusion and insanity appear to have been viewed as convertible terms. Considering, however, *delusion* to be a test of insanity, have the judges uniformly regarded it as *the* standard of responsibility in criminal proceedings? In the case of Overston, Mr. Justice Maule altogether repudiated the test; and in the celebrated Bainbrigge case, Lord Campbell admitted, that "*mania may exist without delusion.*" Need I advance an argument in corroboration of Lord Campbell's dictum, or in opposition to the dogmatic and bold assertion of Lord Denman? It is allowed as the result of the collective experience of those competent to give an opinion upon the matter—that positive, dangerous, and irresponsible insanity, is often seen unassociated with any *manifested* delusive impression, or *appreciable* hallucination.

The legal test that next presents itself for consideration is, the presence or absence of a *motive* for the commission of the crime.* Dr. Prichard observes, " The act of homicidal insanity is different in its nature and moral causes from that of murder. Men never commit crimes without some motive; the inducement which leads them to an atrocious act is of a kind which other men can appreciate and understand, though they do not sympathize with them.

with respect to facts in the natural sciences, which admitted of being verified by easy and simple observation and experiment."—*G. C. Lewis's Essay.*

* " It must for ever be remembered that with motives merely the legislator and the magistrate have nothing to do; and that actions and external facts, as the ends or objects of motives, are the only legitimately cognisable subjects of human tribunals. *Actus non facit reum nisi mens sit rea,* is a rule of reason and justice not less than of positive law."—*On the Principles of Circumstantial Evidence.* By William Wills. 1850.

Jealousy, hatred, revenge, excite some; others are moved by the desire of plunder—of getting possession of money or property. The act of a madman is for the most part without motive." I have thus stated Dr. Prichard's view rather in detail, because I shall feel it my duty to express an opinion adverse to that which this physician entertained. As a test of criminality, I consider the one just propounded not only as unsafe, but as unphilosophical. On the trial of Francis for shooting the Queen, this plea was urged in favour of the prisoner; but what did the then Solicitor-General urge respecting its inapplicability? "This doctrine about motive is of a most dangerous character, and must be very guardedly received. It is very difficult for you (the jury), very difficult for any well-regulated mind not accustomed to contemplate the workings of iniquity—to discover the motives for crime. What motive instigated the execrable assassin in Paris, who shot at his king, and deluged the streets with blood by means of his infernal machine? Did any one ever hear propounded, in a court of justice, a doctrine that would lead to so much dangerous consequences to society, as that you must ascertain the motive before you convict of the crime?" Is this a test, I ask, that can safely be relied upon in all cases of criminal insanity? Are not the insane often impelled to the commission of acts of violence and murder *by the same motives, feelings, and passions, that influence and regulate the conduct of sound, healthy, and rational minds?* There cannot be any difference of opinion upon this point among those practically acquainted with criminal lunatics, and with the characteristics of mental derangement. It would be monstrously cruel and unjust to apply such a test in criminal cases. Were such to be our standard of appeal, great crimes would escape unpunished, and persons indisputably insane and irresponsible would often (to our great reproach) suffer the extreme penalty of the law!

A patient who was confined in the Manchester Lunatic Asylum had been subjected to very cruel treatment by the person who had the care of him. He consequently attacked the attendant, and killed him. He related, with great calmness and self-possession, the particulars of the transaction to the physician of the asylum. He said, "The man whom I stabbed richly deserved it. He behaved to me with great violence and cruelty; he degraded my nature as a human being; he tied me down, handcuffed me, and confined my hands much higher than my head with a leathern thong; he stretched me on the bed of torture; after some days he released me. I gave him warning, for I told his wife I would have justice of him. On her communicating this to him, he came to me in a furious passion, threw me down, dragged me through the court-yard, thumped me on the breast, and confined me in a dark and damp cell. Not liking this situation, I was induced to play the hypocrite. I pretended extreme sorrow for having threatened him, and by an affectation of repentance, prevailed on him to release me. For several days I paid him great attention, and lent him every assistance. He seemed much pleased with the flattery, and became very friendly in his behaviour towards me. Going one day into the kitchen, where his wife was busied, I saw a knife; this was too great a temptation to be resisted; I concealed it about my person, and carried it with me. For some time afterwards the same friendly intercourse was maintained between us; but as he was one day unlocking his garden-door, I seized the opportunity, and plunged the knife up to the hilt in his back." He always mentioned this circumstance with peculiar triumph, and his countenance, a most cunning and malignant one, became highly animated at the conclusion of the story.*

The following case in point excited much interest some years back, in Philadelphia. Eighteen years previously to the commission of the crime, a confectioner of the name of Wood had come from England; had carried on his trade first in New York, and then in Philadelphia; had realized money, and acquired a respectable character. He had an only daughter, whom he was desirous of advancing into a higher station by marriage. But he himself was not in genteel society; yet he restrained her from associating with persons of her own condition; and she therefore had no freedom in any circle. She assisted him in keeping his shop, one of the first of its kind in Philadelphia. A young man of inferior habits and station made love to her, and persuaded her to leave her father's roof and marry him. She was absent only one night, when she returned home, and confessed she was a married woman. Her father became violently and passionately

* Dr. Haslam.

excited; he drank a large quantity of rum; and, under the combined influence of disappointed ambition, rage, and intoxication, he shot his daughter with a pistol. He did not attempt to escape. When he became sober and free from excitement, he had no knowledge of his crime. He was tried for the murder. His counsel pleaded insanity, and proved previous mental aberration; but, in his defence, he mainly relied on the shock given to his feelings by his daughter's conduct having produced a real insanity preceding the homicide. A verdict of lunacy was recorded. If this case had occurred in England, it is questionable whether he would have been acquitted.

It has been proposed, that the question of legal responsibility should be determined by the fact, whether the party, when he committed the offence, knew that he was acting in opposition to those generally-received and recognised moral obligations which are supposed to govern and influence sane, rational, and christian minds. The question which the jury has to consider, to use the language of one of the most distinguished and enlightened British judges that ever adorned the bench, is, "Was the prisoner conscious that he was committing a crime against the laws of God and nature?"*

In considering the value of this test, we are bound to remember that there is a class, happily for themselves and for society insignificant in a numerical point of view, who repudiate the idea of a Divine law regulating their actions and as binding upon their consciences, and who deny the existence of a Supreme Being. I readily admit that in all important matters of legislation we are not justified in considering the anti-christian or heterodox opinions of small sections of the community. All our legislative enactments are rightly based upon the assumption that the great mass of mankind worship a Supreme Being, and implicitly recognise the doctrine of a Divine revelation; nevertheless, if we have a legal test or standard to which we can refer, it should be *catholic* in its character, and be susceptible of universal application. Imagine a person arraigned for the commission of a capital crime. The plea of insanity is urged in his defence. In expounding the law, the judge informs the jury, that the question of responsibility in connexion with insanity rests upon the fact

* Lord Lyndhurst.

whether the prisoner had at the time a consciousness of his having deviated from the law of God. Was he sensible of this? If so, he is to be considered amenable to justice, and must expiate his crime upon the gallows. I can conceive, after such a legal exposition, the prisoner making a declaration of his being by virtue of his principles *placed beyond the jurisdiction of such a test*, and maintaining that he could not morally, legally, or logically be considered to be conscious of violating laws that in reality he never believed to exist. I will admit that this may be considered to be an extreme case. I merely cite it with the view of establishing my position, that there is no legal test yet propounded applicable, or which could be indiscriminately applied, to *all* criminal cases of insanity.

Among the other judicial standards or criteria of insanity, is that recommended by the late Lord Chief Justice Tindal. I refer to his suggestion to apply the test of the knowledge of "right and wrong" to every case of crime alleged to be associated with and the result of insanity, and upon its existence or non-existence to determine the presence of legal responsibility. Lord Chief Justice Mansfield says, in reference to this test,* "The law is extremely clear. If a man was deprived of all power of reasoning, so as not to be able to distinguish whether it was right or wrong to commit the most wicked or the most innocent transaction, he could not certainly commit an act against the law. Such a man, so destitute of all power of judgment, could have no intention at all. In order to support this defence, however, it ought to be proved by the most distinct and unquestionable evidence that the criminal was incapable of judging between right or wrong. It must in fact be proved, beyond all doubt, that at the time he committed the atrocious act with which he stood charged, he did not consider murder was a crime against the laws of God and nature. There was no other proof of insanity which could excuse murder or any other crime. There were various species of insanity. Some human creatures were void of all power of reasoning from their birth; such could not be guilty of any crime. There was another species of madness, in which persons were subject to temporary paroxysms, in which they were guilty of acts of extravagance; this was called lunacy

If these persons committed a crime when they were not affected with the malady, they were, to all intents and purposes, amenable to justice. So long as they could distinguish good from evil, so long would they be answerable for their conduct. There was a third species of insanity, in which the patient fancied the existence of injury, and sought an opportunity of gratifying revenge by some hostile act. If such a person was capable, in other respects, of distinguishing right from wrong, there was no excuse for any act of atrocity which he might commit under this description of derangement. The witnesses who had been called to support this extraordinary defence had given a very singular account, in order to show that at the time of the commission of the crime the prisoner was insane. What might have been the state of his mind some time ago, was perfectly immaterial. The single question was, whether at the time this act was committed, he possessed a sufficient degree of understanding to distinguish good from evil, right from wrong, and whether murder was a crime not only against the laws of God, but the law of his country."*

It has been a question with metaphysicians, whether, *abstractedly* considered, there are conditions or states to which the terms "right and wrong" can, with strict philosophical precision, be applied. Are not these general terms, it is urged, merely significant of the resemblance of various particular actions which agree in exciting in the mind certain feelings that are analogous? In different phraseology, are not the terms "right and wrong" general expressions indicative only of analogous relations which certain actions bear to certain emotions? Dr. Hutchinson (who has, perhaps, taken a more ultra view of this question than any other metaphysician) says, "There is no right or wrong, virtue or vice; but there are agents whose actions cannot be contemplated by us without an emotion of approbation or disapprobation; and all actions, that is to say, all *agents* that agree in exciting moral feelings, which are thus analogous, we class together as virtuous or vicious from this circumstance of felt agreement alone.

* "They could only know one kind of right and wrong; the right is, when you act according to law; and the wrong is, when you break it. Distinguishing right from wrong, meant a knowledge that the act the person was about to commit was punishable by the law."—Lord Brougham: *Speech in the House of Lords.*

The similarity of the emotions which we feel in these particular cases, is thus all to which we owe the notions or ideas of right or wrong, virtue and vice." Dr. Brown, in commenting upon this passage, observes, "that right and wrong signify nothing in the objects themselves. They are words expressive only of relation, and relations are not existing parts of objects, or things to be added to objects, or taken from them. There is no right or wrong, merit or demerit, *existing independently of the agents who are virtuous or vicious*."*

I allude to these generally received metaphysical *dicta*, not because I would be guilty of so gross an absurdity as to deny the existence of such principles of action, but because I infer from the particular and special reference made to this legal test, that it is supposed these conditions are easily appreciable and almost tangible states, to which in complex and obscure cases the jurist and psychologist might at once appeal for an immediate and certain solution of their difficulty.

Before this test can be admitted as a safe standard in questions of moral and legal responsibility, it will be necessary to establish an infallible rule, by which we may be able to trace accurately the distinction between right and wrong. Moral philosophers, men of science, theologians, political and social economists, philosophers and statesmen, are unfortunately very much at variance upon many apparently self-evident, and first principles, relating to their respective departments of inquiry. Need I refer to the great discrepancy of opinion existing among different religious denominations, each sect maintaining its own *dicta* to be "right," and the views of other sections of the religious world to be "wrong." The Roman Catholics consider themselves to be right, and the Protestants to be wrong, and *vice versâ*. A few of the Quakers, objecting upon principle to all war, on the ground of its not being sanctioned by scripture, and from a conviction of its being morally wrong, resolutely refuse to pay a war-tax. We differ from the Quaker, and entertain the opinion, that, under certain circumstances, war is right and justifiable. A large section of the religious community denounce infant baptism as "wrong;" a still more important body think it "right." There are many who repudiate the hierarchy and priesthood, from a conviction of their anti-scrip-

* On the Philosophy of the Human Mind. By Dr. T. Brown.

tural origin, character, and tendency. If we turn to the political and scientific world, we find large bodies of intelligent and reflecting men holding opinions diametrically at variance with each other, and advocating the most opposite and irreconcilable views of the same question, and contending most heroically for the truth of their own individual and sectional opinions, from a persuasion —an unalterable conviction—of their being "right," and the sentiments of their adversaries, "wrong." The *right* of *to-day*, in matters of theology, philosophy, and science, may be the *wrong* of *to-morrow;* and what is now "lawful," may, in the course of a short parliamentary session, be made illegal by the introduction of new statutes! Analyzing this much-eulogized legal test as metaphysicians, as medical philosophers, and as men of the world, are we not compelled to pronounce it to be worthless, and practically inapplicable? Our views of "good and evil," "right and wrong," "lawful and unlawful," must necessarily be dependent upon, and fluctuate in obedience to, temperament, caste, climate, progress of civilization, education, knowledge, early training, and example. If there be within us an innate principle termed "conscience," acting *independently of the judgment*—and existing as a separate agent or faculty of the mind (which many metaphysicians and theologians deny), unerringly suggestive to us of a knowledge of "right and wrong," is not this moral sense or instinct often destroyed by adventitious circumstances, its perceptions deadened, paralyzed, or perverted?

Considering this legal test of criminality apart altogether from the metaphysical objections to which it is amenable, I maintain, that it never can be safely depended upon, in all cases of insanity. It is a notorious fact—a matter of every-day occurrence, and in accordance with the experience of those conversant with the phenomena of lunacy,—that the insane—the positively and undeniably insane—like many rational persons, often

"Know the 'RIGHT,' and yet the 'WRONG' pursue,"

and frequently act in direct opposition to their own clear and unmistakable convictions of what is "right and wrong," "good and evil," "lawful and unlawful." Many a maniac has committed a crime of great atrocity, with a full, unfettered, and unclouded consciousness and knowledge of its unlawfulness, its sinfulness, its criminality, and of the legal penalties to which he is, by his actions, exposing himself. A lunatic has manifested an intense and morbid desire for death; not being suicidally disposed, he endeavours to effect his purpose by sacrificing the life of another: he designedly brings himself within the pale of the law, that he may compel others to do what he has not the courage of accomplishing himself. How absurd, cruel, and unjust it would be to apply the test of a knowledge of what is lawful or unlawful to such a case?

An intriguing, unruly, vicious lunatic was detected with a piece of iron which he had contrived to shape like a dagger, with a handle fixed firmly in it. Upon being interfered with, he became excited, abusive, and violent. He was placed under restraint; after uttering the most awful imprecations, he exclaimed to his attendant, "I'll murder you yet; *I am a madman, and they cannot hang me for it!*"

When Martin set York Minster on fire, a conversation took place among the inmates of a neighbouring lunatic asylum, having reference to this general topic of remark and discussion. The question argued was whether Martin would suffer the extreme penalty of the law for his crime. Various were the opinions expressed. In the midst of the conversation, one patient, apparently as mad as the rest, exclaimed, "He (Martin) will not be hanged." "For what reason?" interrupted several voices. "They cannot hang him," replied the lunatic, "*he is one of ourselves.*" Of what value is this legal test, if applied to such cases? Before this can be recognised as a safe standard, it will be necessary for British jurists to lay down for their *own* guidance certain fixed and unalterable principles of jurisprudence. Is it not a notorious fact, that on apparently clear and well recognised points, lawyers of eminence have arrived at the most opposite conclusions? One court reverses the judgment of an inferior tribunal, and one distinguished jurist overrules the decision of his predecessor. As long as able judges differ among themselves upon what may be termed *first principles of law*, it will be unreasonable to expect that *we* should prostrate ourselves before the legal test which I have been analyzing.

Dr. Mittermaier, a German jurist, maintains that two conditions are required to constitute that freedom of will which is essential to responsibility—viz., a knowledge of good and evil, and the facility of choosing between them. The knowledge of good and evil will require, first, that knowledge of one's self by which we recognise our personal identity, and refer our acts to ourselves; secondly, acknowledgment of the act itself—*i. e.*, of its nature and consequences; thirdly, a knowledge of the relations of the act both in regard to men and measures; fourthly, a knowledge that the act in question is prohibited either by the moral or the statute law. He rebukes the English jurists for their rigid adherence to the antiquated doctrine, that whoever can distinguish good from evil, enjoys freedom of will, and retains the faculty, if he chooses to use it, of framing his actions to the requirements of the law. The true principle, according to this authority, is to look at the personal character of the individual whose responsibility is in question; to his grade of mental powers; to the notions by which he is governed; to his views of things; and finally to the whole course of his life, and the nature of the act with which he is charged. A person who commits a criminal act, being fully cognisant of the nature of the laws, and of the punishment to which he is exposing himself, may yet be of insane mind. The true test of irresponsibility should be, not whether the party accused was aware of the criminality of his actions, but whether *he has lost all power of control over his actions.*

As the plea of insanity is one of the most important that can be urged in a court of justice in extenuation of crime, it should never be had recourse to except in clear and obvious cases, in which little or no doubt can be entertained, not only of the existence of mental derangement, but of derangement of such a *kind*, and to such a *degree*, as to justify the immediate admission of the fact, and the necessary and consequent acquittal of the prisoner. The utmost vigilance and jealous caution should be exercised in all inquiries of this nature; and medical men, considered specially competent to the elucidation of such intricate psychological phenomena, should be particularly guarded in sanctioning, by their authority, the plea of insanity, exhibiting, upon all occasions, a fear lest their opinions should be made available for the purposes of shielding great criminals from the just and legal penalties awarded for the commission of crime.

The reflecting portion of the public and profession naturally place a high value upon the experience, testimony, and judgment of men whose peculiar studies and opportunities enable them to obtain a practical insight into morbid aberrations of mind. If it be found that men of position and ability are disposed to be lax in the use of this important plea, a reaction will inevitably ensue, and cases of this character will be left exclusively to the adjudication of the judicial tribunals, medical evidence being entirely ignored in our courts of law.

In forming an opinion of the criminal as well as the civil responsibility of any case of alleged insanity, it is very essential, with the view of our arriving at right results, that we should make a just and scientific distinction between the actions of a *naturally* eccentric, ill-regulated, perverse, and wicked mind, and the mental disturbance, perverseness, caprice, vice, extravagance of conduct, ungovernable passion, sullenness of disposition, and melancholia, consequent upon physical disease of the *sensorium*, or organs in close pathological relationship with it, implicating the healthy action of thought. There is a *normal* and *natural* eccentricity, a *healthy* mental idiosyncrasy, caprice, and feeling, distorted and perverted affection, disposition to acts of cruelty, vice, brutality, *existing independently of that irregularity and disturbance in the operations of the intellect—those perversions of the affections and madness of conduct, the clear, unmistakable, and undoubted consequence of a* DISEASED MIND. As a man may have *natural physical*, so may he exhibit a *connate mental* defect, apart altogether from actual cerebral, and consequent mental disease. It should never be forgotten that there is always floating upon the surface of society a large body of strange, wayward, intemperate, eccentric persons, criminally and viciously disposed, subject to every bad passion, impulsive in all their movements, addicted to habits of debauchery, who lead a kind of animal life; whose mode of existence appears fully to realize Lady Morgan's somewhat illiberal conception of the character of the modern Italians—

> "Who eat, drink, and sleep. What then?
> Who sleep, drink, and eat again."

There is a healthy and *natural* melancholy, and a *diseased* depression of spirits. There is a species of drunkenness which is *not* insanity, and there is a form of mental derangement solely indicated by inveterate and uncontrollable habits of intemperance.

There is a brutality existing irrespectively of lunacy, and violence of conduct, and cruelty of disposition, clearly the effects of a morbid mental condition. There is a natural, and, speaking medico-psychologically, a healthy improvidence, impetuosity of temper, and vice, which should not be confounded with *ab*normal and diseased states of the affections, passions, appetites, and propensities. The melancholia—the sullen gloom—the moroseness of real life (which is *not* "alienation of mind"), is well described by an able metaphysician:*—"It disposes the person to acts of unkindness, and makes him the slave of every bad passion; it produces a fretfulness in all the daily and hourly intercourse of life; it produces a domestic tyranny which brings, alas! with it a train of heartburnings and bitterness. This melancholy temper is poisonous to the happiness, not only of the individual, but of all that are brought within the circle of its baneful influence."

Beattie's "Minstrel" is described as one of those half-cracked, half-witted, sombre, clever, sullen, eccentric, melancholy youths; the *type* of thousands who are daily mixing in society, and whose condition might easily, upon a superficial examination, be confounded with insanity, and whose state of mind would certainly, by some, be considered "unsound," were they guilty of any *overt* act of sufficient importance to call public and professional attention to their moral and legal responsibility.

> "Silent when glad; affectionate, though shy;
> And now his look was most demurely sad,
> And now he laughed aloud, yet none knew why,
> The neighbours stared and sighed, yet bless'd the lad;
> Some deemed him wondrous wise, and some believed him mad."

I cannot conceive a position of graver responsibility than that assumed by the medical witness when called upon in a court of justice to give evidence in criminal cases. Let me earnestly entreat him, before discharging these solemn duties, to make himself master of all the facts of the case. He should not assume for granted the representations of those anxious to establish the insanity of the criminal; were he to do so, he would occasionally be sadly deceived. He should never forget that he has a *public* as well as a *professional* duty to discharge; and he is bound, as a citizen of the state, as well as a member of an important and

* *Philosophy of the Human Mind*, chap. on "Immediate Emotions." Dr. T. Browne.

learned section of society, to protect himself from the possibility of being deceived as to the facts of any given case presented to him for his opinion. He must not permit his feelings to overpower and interfere with the free and unclouded operations of his judgment.

Under these circumstances, every possible influence will occasionally be exercised to induce the witness to adopt an opinion favourable to the prisoner. He will perceive the necessity of patiently investigating the case itself, and will not be satisfied with one or two interviews with the alleged lunatic. He must obtain from the criminal an account of the act with which he may be charged, and his reasons for committing it; he will also acquire from his relatives, friends, and companions, an insight into his former mode of life—his habits of thought—his prior state—the peculiarities of his disposition—whether there exists in the case an hereditary predisposition to insanity; and other circumstances likely to elucidate the actual state of the mind at the time when the alleged offence was perpetrated. Great perseverance and ingenuity are often required before the truth can be elicited. In these cases, the crime is occasionally committed during a paroxysm of transient insanity; the mind manifesting no symptom of derangement after the perpetration of the offence. Again, a lunatic has been known to commit murder in a fit of frenzy, his sudden arrest and committal to prison temporarily restoring the mind to its healthy balance. A man has been guilty of a capital crime; has been seized and sent to prison, and has, from remorse, or a sense of horror at his position, suddenly become insane; his derangement only exhibiting itself after his arrest. Persons have been known to commit the crime of murder whilst in a state of somnambulism, and also during that half-unconscious condition between sleeping and waking. Cases of this description are extremely perplexing to medical jurists. If it can be satisfactorily proved that the person perpetrated the murder whilst in this state—if the fact be unequivocally established—then, I conceive, it ought to be considered as a good exculpating plea. It should never, however, be forgotten, that these cases are easily simulated. Examples of this character are recorded by medical writers. A person has been suddenly roused by a frightful dream, and, whilst under its influence, has been known to take away human life. Suicide has been committed under analogous circumstances. A person, apparently well, has gone to bed without manifesting the slightest tendency to self-destruction; he has awoke suddenly and destroyed himself. A case, illustrative of this fact, is on record. It is as follows: "An old lady residing in London awoke in the middle of the night, went down stairs, and threw herself into a cistern of water, where she was found drowned." It was maintained that the suicide was the result of certain mental impressions conjured up in the mind during a dream. Dr. Pagan refers to the following interesting case, to prove that murder may be committed by a person when under the effects of a frightful vision.

Bernard Schedmaizig suddenly woke at midnight; at the moment he saw a frightful phantom, or what his imagination represented as such—a fearful spectre! He twice called out, "Who is that?" He received no answer. Imagining that the phantom was advancing upon him, and having altogether lost his self-possession, he raised a hatchet which was beside him, and attacked the spectre: it was found that he had murdered his wife!

A pedler, who was in the habit of walking about the country armed with a sword-stick was awakened one evening, while lying asleep on the high road, by a man suddenly seizing him, and shaking him by the shoulders. The man, who was walking by with some companions, had done this out of a joke. The pedler suddenly woke, drew his sword, and stabbed the man, who soon afterwards died. He was tried for manslaughter. His irresponsibility was strongly urged by his counsel, on the ground that he could not have been conscious of his act in the half-waking state. This was strengthened by the opinions of medical witnesses. He was, however, found guilty.* The murder, in this instance, may have been the result of passion. We have no evidence to the contrary.

In criminal cases, should the witness be interrogated as to the alleged lunatic's consciousness of right and wrong, or as to his knowledge that he was violating the law of God and man at the moment when the crime was committed, I would strongly suggest that he should, unless the case be one of obvious lunacy,

* British and Foreign Medical Review.

decline answering the question. The witness may have a clear and positive opinion as to the existence of insanity; but how can he, in every case, solve the question as to the lunatic's ability to distinguish accurately between good and evil, right and wrong, lawful and unlawful? Dr. Haslam says, when alluding to this point, that "It is not the province of the medical witness to pronounce an opinion as to the prisoner's capability of distinguishing right from wrong. It is the duty of the medical man, when called upon to give evidence in a court of law, to state whether he considers insanity to be present in any given case, not to ascertain the quantity of reason which the person imputed to be insane, may or may not possess. If it should be presumed that any medical practitioner is able to penetrate into the recesses of a lunatic's mind at the moment he committed the outrage; to view the internal play of obtruding thoughts and contending motives; and to depose that he knew the good and evil, right and wrong, he was about to commit,—it must be confessed, that such knowledge is beyond the circuit of our attainment. It is sufficient for the medical practitioner to know that the person's mind is deranged, and that such a state of insanity will be sufficient to account for the irregularity of his actions; and that in a sound mind the same conduct would be deemed criminal. If violence be inflicted by such a person during a paroxysm of rage, there is no acuteness of metaphysical investigation which can trace the succession of thoughts, and the impulses by which he is goaded for the accomplishment of his purpose."

In many cases the plea of insanity is entirely based upon the assumption that the prisoner is "*morally insane.*" It is much to be lamented that the term "moral insanity" was ever introduced by Dr. Prichard into our psychological nomenclature. The phrase is generally repudiated in our courts of law; it has given rise to much cavilling and disputation, and its adoption has unfortunately exposed the profession to great odium and obloquy; and has, I think, very materially damaged the moral weight of medico-legal testimony. It has been asserted, that the term is used with the view of protecting the criminal from just punishment, and of shielding vice, extravagance, malignity, debauchery, cruelty, crime, and brutality, from the natural emotions of horror and disgust with which such actions should be contemplated by every right-thinking and well-constituted mind. "Moral insanity!" I might conceive the judge to exclaim; "I will not listen to such an excuse—to such a plea—to such evidence! I will not sit here, and, whilst administering justice, permit the great truths of science to be thus perverted and abused, with the view of destroying the practical application, and beneficial and conservative operation, of the criminal law of the land!"

Let us consider the subject of "moral insanity," or, as Pinel terms it, "*emportement maniaque sans délire*," not only *pathologically*, but *metaphysically*. All authorities agree in opinion, that the specific characteristics of this form of derangement are dependent upon a lesion of the affective or motive powers of the mind, apart altogether from disorder of the *intellectual* faculties, or powers of ratiocination. In the first place, I would ask, whether the disease so designated is *purely an affection of the moral faculties;* and whether, as metaphysicians, we are justified in drawing so palpable a line of demarcation between those faculties of the understanding that reason, judge, compare, reflect, and those that supply motives to the reason, and are termed, by metaphysicians, the *active* principles of the mind?

Viewing the question under review *pathologically*, I ask whether, in cases of insanity which are represented to consist in lesions of the will—in ungovernable impetuosity of temper—loss of self-control—perversion of the affections and propensities—cases in which the mental alienation is manifested more in *conduct* than in *ideas*—where the delirium is apparently confined to the *actions* and *moral sentiments;*—whether in this form of mental derangement, the intellectual, the reasoning, and reflective powers are not more generally disordered than we have hitherto admitted? In many instances of mental disease, considered as uncomplicated illustrations of moral insanity, the malady *is not confined to the affective or motive faculties.* I do not maintain that such is *apparent* in every case of impairment of the moral sense or motive power; but I have detected the intellectual aberration in many cases brought under my observation as instances of pure derangement of the conduct, propensities, passions, appetites, and moral affections. In nearly all of them we may, upon a close logical analysis, perceive co-existing with the moral disorder, a derangement of those powers of the mind by which we compare facts with each other, and mental impressions with external things; to speak with metaphysical exactness, and philosophical as well as philological precision—by which we *appreciate the perception of relation.* If we carefully investigate the cases quoted by Pinel, Esquirol, and Prichard, and referred to as *types* of moral insanity, we are irresistibly led to the conclusion, that the malady, as described by these authorities, was not in *any one case restricted to the affective or motive powers of the understanding.* The faculties of judgment, reason, and comparison, are represented in this form of insanity to be healthy and *intact.* Apparently, upon a superficial examination, they may be so; but do not the "tyrant passion"—predominant vice—overpowering emotion—loss of self-respect—brutality of conduct—prostration of all the more refined sensibilities of the mind—uncontrollable impulse—impetuous will—and the suicidal or homicidal idea, during the crisis of the paroxysm, *and contemporaneously with the commission of the act,* dethrone reason, and paralyze the operations of the judgment? Do not violent and ungovernable temper, impulsive emotion, and unreasonable conduct, leading to overt acts of what are termed moral insanity, suspend the exercise of the will, and interfere with the healthy balance or equilibrium of the *intellectual* faculties? In cases where the faculty of volition appears to be suspended, and the patient is unhappily the willing and facile slave of every wicked, sensual appetite and vicious propensity, and is guilty of most extravagant conduct—are, I repeat, the powers of *judgment, reason,* and *comparison,* the more exalted and intellectual functions of the mind, entirely free, unclouded, unfettered, and in a healthy state of activity? Is the "moral maniac" capable of pursuing an ordinary and healthy process of induction, and competent to exercise the powers of reason, comparison, and reflection, *quoad* the specific features of his so-termed "moral" disease? He may be apparently of sound understanding, able to solve with great rapidity a difficult mathematical problem; have great capacity for the ordinary business of life; may converse with ease upon points of science, art, and philosophy; and astonish the world by the tenacity of his memory, the vividness of his fancy, the playfulness of his satire, the brilliancy of his wit, and the majesty and sublimity of his eloquence—all these elevated states of mind are compatible with *latent delusive ideas and intellectual disorder.** Lord Brougham makes some pertinent remarks on this subject. When applying his able powers of philosophical analysis to this question, his lordship observes: "We cannot with any correctness of language speak of general or 'partial' insanity; but we may most accurately speak of the mind exerting itself in consciousness without cloud or imperfection, but being morbid when it fancies; and so its owner may have a diseased imagination, or the imagination may be diseased, and yet the memory may be impaired, and the owner be said to have lost his memory. In these cases we do not mean that the mind has one faculty, as consciousness, sound, whilst another, as memory or imagination, is diseased; but that the mind is sound when reflecting upon its own operations, and diseased when exercising the combination termed imagination, or casting the retrospect called reflection." Then again, as to what is termed impulsive insanity, a form of disease generally considered to be unassociated with derangement of the ideas, I would ask, is it a fact that these cases are invariably unaccompanied by delusive impressions, or by a disturbance of the reasoning faculties? Admitting the existence of a morbid impulsive propensity, does it become absolutely irresistible and uncontrollable except during a crisis of delirium? It has been maintained, that at the moment of the impulsion an intellectual perturbation and positive derangement of ideas occurs. "We believe," says a French writer, "that the doctrine of a temporary insanity, of a sudden eclipse of the reason at the time of the act, is a safer and more philosophical doctrine than the hypothesis of modern medical jurists, who assert that no monomania, whether homicidal, suicidal, or incendiary, can compel to the consummation of the act, without insanity in the ordinary acceptation of

* In many cases, designated as illustrations of moral insanity, I feel assured that undetected and unrecognised delusions often actually exist, influencing the conduct of the patient. I could narrate several instances of the kind. M. Marc mentions the case of a man, who for many years had been in the habit of licking the walls of the apartment with his tongue, until he had actually worn away the plaster. No one could imagine what was the cause of this perseverance in so painful and disgusting a habit, until one day in the author's presence he confessed that he tasted and smelt the most delicious fruit on the walls.—(p. 119.)

the term, or intellectual disturbance. We repeat, that we cannot admit this theory or principle of monomania with irresistible desire, and without delirium during the act, because it appears to us to be dangerous, inasmuch as it suspends the course of free-will, is destructive of the morality of human actions, and tends to favour impunity for crimes. For if the impulse be irresistible, and is unaccompanied by delirium during the act, what becomes then of free-will? In our minds, the disturbance of the reason will always be more comprehensible and conformable to the common-sense of mankind than a perversion of the will without delirium."

Having considered this subject pathologically, I would briefly analyze it *metaphysically.* In using the words "mind," "intellect," "understanding," we employ abstract terms to denote an aggregate condition of all the phenomena of intelligence, to describe the manifestations of *one and an indivisible essence.* In classifying, for the convenience of philosophical investigation, the mind into separate and distinct powers or faculties, emotions or passions, are we not oblivious of the fact, that this arrangement, classification, order, division, and subdivision, are essentially arbitrary, and that the principle, essence, and substratum of mind, is in itself a unit, and incapable of being subjected to such divisions and classifications? Many of the so-termed faculties of the mind, the emotions and passions, which are spoken of as independent and distinct powers, are obviously only modifications of, or different modes of being or manifestations of, ONE *particular mental condition or state of intellectual relation.* "We cannot map out the mind as we can a country or a county, assigning to each town, province, or state, its separate controlling and free sovereignty. We are not justified in converting each faculty into a little 'independent mind,' as if the original mind were like that of the polypus, which, according to naturalists, may be cut into an almost infinite number of parts, each of which becomes a polypus, as perfect as that from which it was separated."* "I suspect," says Locke, "that this way of speaking of the faculties has misled many into a confused notion of so many distinct agents in us, which had their several provinces, and did command, obey, and perform several actions as so many

* Browne.

distinct beings; which has been no small occasion of wrangling, obscurity, and uncertainty, in questions relating to them." "The mind," says another eminent authority, "is formed susceptible of certain affections; these states or affections we may generalize more or less, and, according to our generalization, may give them more or fewer names." "But," he continues, "whatever may be the extent of our vocabulary, the mind itself is as independent of these transient designations as *He* who fixed its constitution—still continues to exhibit the same unaltered susceptibilities which it originally received; as the flowers which the same Divine Author formed, spring up in the same manner, observing the same seasons, and spreading to the sun the same foliage and blossoms, whatever be the systems and the corresponding nomenclature, according to which the botanists may have agreed to record and name their tribes. The great Preserver of Nature has not trusted us with the dangerous power of altering a single physical law which *He* has established, though he has given us unlimited power over the *language* which is of our own creation." May we not apply the same argument to the phenomena of life? We observe the principle of vitality manifested through different physical *media;* but whatever may be the character of the material tissue, or the special function of the organic structure through which life reflects its powers, we, as spiritual physiologists, maintain that these manifestations are only different *modes* or *states* of development of *one and the same principle;* that the life that manifests itself through the brain, lungs, stomach, and the heart, is identical and homogeneous in its nature and essence; the peculiarity of the physical organization affecting, as it undoubtedly does, its mode of being or action. Applying this metaphysical doctrine to the subject now under consideration, it must be evident, that in all the varied phenomena of insanity the same identical essence or principle is affected; that, without any exceptions, THE MIND—using this term in its liberal and philosophical acceptation—is in a state of disorder. I would, however, protect myself from the imputation of repudiating the great discovery of Gall, or of holding, with the spiritualists, that the principle of thought is susceptible of actual disease, apart from any abnormal state of the cerebral tissue. In all cases of mental derangement, the *manifestations* of the mind, and not the mind *itself,* are implicated; or, to speak with a strict regard to the principles of cerebral pathology, the *physical media*, or different portions of nervous matter through which the intellect is developed, are diseased, and, as a necessary consequence, the principle of thought is disordered or deranged in its operations. As there appears a determination to discountenance the use of the term "moral insanity," I would advise the witness to avoid, upon all occasions, an ostentatious and unnecessary application of the phrase. If called upon to give evidence in cases of insanity, involving apparently the healthy action of the motive and *affective* powers, I would recommend the witness, when asked to state his opinion of the condition of the mind and the degree of responsibility in cases of this nature, to speak of the disorder as one implicating the normal state of the *mental principle*. In reply to the interrogatory—"Do you consider the prisoner at the bar of sound mind, and a responsible agent?"—I would suggest to the witness the safety of answering, to the best of his judgment, either affirmatively or negatively; bearing always in recollection, that in all phases and degrees of insanity, whatever form it may assume, *one* and the *same essence* is involved in the disturbance—that all are, strictly speaking, AFFECTIONS OF THE MIND.

(*To be concluded in our next Number.*)

